# Recent Progress in the Development of Aromatic Polymer-Based Proton Exchange Membranes for Fuel Cell Applications

**DOI:** 10.3390/polym12051061

**Published:** 2020-05-06

**Authors:** Raja Rafidah R. S., Rashmi W., Khalid M., Wong W. Y., Priyanka J.

**Affiliations:** 1 School of Engineering, Taylor’s University, Subang Jaya 47500, Malaysia; rafeedahrosset@gmail.com; 2 Department of Chemical Engineering, School of Energy and Chemical Engineering, Xiamen University Malaysia, Sepang 43900, Malaysia; 3 Graphene and Advanced 2D Materials Research Group (GAMRG), School of Science and Technology, Sunway University, Subang Jaya 47500, Malaysia; priyankaj@sunway.edu.my; 4 Fuel Cell Institute, UniversitiKebangsaan Malaysia, UKM Bangi, Selangor 43600, Malaysia

**Keywords:** fuel cells, aromatic-based, polymers, proton exchange membranes, modifications

## Abstract

Proton exchange membranes (PEMs) play a pivotal role in fuel cells; conducting protons from the anode to the cathode within the cell’s membrane electrode assembles (MEA) separates the reactant fuels and prevents electrons from passing through. High proton conductivity is the most important characteristic of the PEM, as this contributes to the performance and efficiency of the fuel cell. However, it is also important to take into account the membrane’s durability to ensure that it canmaintain itsperformance under the actual fuel cell’s operating conditions and serve a long lifetime. The current state-of-the-art Nafion membranes are limited due to their high cost, loss of conductivity at elevated temperatures due to dehydration, and fuel crossover. Alternatives to Nafion have become a well-researched topic in recent years. Aromatic-based membranes where the polymer chains are linked together by aromatic rings, alongside varying numbers of ether, ketone, or sulfone functionalities, imide, or benzimidazoles in their structures, are one of the alternatives that show great potential as PEMs due totheir electrochemical, mechanical, and thermal strengths. Membranes based on these polymers, such as poly(aryl ether ketones) (PAEKs) and polyimides (PIs), however, lack a sufficient level of proton conductivity and durability to be practical for use in fuel cells. Therefore, membrane modifications are necessary to overcome their drawbacks. This paper reviews the challenges associated with different types of aromatic-based PEMs, plus the recent approaches that have been adopted to enhance their properties and performance.

## 1. Introduction

A solid ion-conducting electrolyte membrane is one of the vital core components in fuel cell systems, namely for the types operating at a temperature range between room temperature and 200 °C, such as Low and High Temperature Proton Exchange Membrane Fuel Cells (LTPEMFCs and HTPEMFCs), Anion Exchange Membrane Fuel Cell (AEMFCs), Direct Methanol Fuel Cell (DMFCs), and Microbial Fuel Cell (MFCs) [1]. The solid electrolyte functions as a separator between the anode and cathode, repels electrons and acts as a barrier between thefuel and oxidants [2,3]. Proton exchange membranes (PEMs) applied in PEMFCs and DMFCs are cationic exchange membranes possessing negatively charged groups (SO_3_^−^, -COO^-^, -PO_3_^2−^, etc.) on the membrane’s polymeric backbone that provide a conducting pathway for cations, normally protons, but reject anions. Conversely, the polymeric backbone of anion exchange membranes (AEMs) for AEMFCs holdspositively charged groups (-NH_2_^+^, -NR_2_H^+^, -PR^+^, etc.) for the transport of anions, such as hydroxides [4]. Achieving high performance in fuel cells requires that these ion exchange membranes have desired characteristics in terms of their electrochemical properties and durability, such asa high proton/anion conductivity (approximately, σ = 0.1 S/cm); low electron conductivity; good resistance to fuel crossover; excellent mechanical, thermal, and chemical strength; acceptable hydrolytic and oxidative stability; and low cost of fabrication and assembly [4,5,6,7,8].

Perfluorosulfonic acid (PFSA)-based polymers, namely the Nafion membrane, have been the standard for fuel cell PEMs due to their high proton conductivity and excellent durability. However, the performance of Nafion is affected by its poor methanol resistance and drop in proton conductivity under high temperatures and low humidity conditions. Additionally, the membrane is also expensive [9,10]. In fuel cell production, the cost of the stack covers 66% of the whole system, and the membrane contributes to 17% of the stack cost for the production of 1000 systems per year (DOE Fuel Cell Report 2017) [11]. Efforts to reduce the cost and improve the performance of each component in the fuel cell stack have been made by researchers in recent years. For membranes, several potential, low-cost alternatives to Nafionhavealready been extensively studied. Among these alternatives to the Nafion membrane are biopolymers based on chitosan, alginates, or cellulose, and the non-fluorinated or partially-fluorinated hydrocarbon polymer membranes with aromatic backbone structures. While biopolymers have advantages in terms of their renewability, durable properties under fuel cell operating conditions, and lower cost, aromatic-based membranes are equally as advantageous, having an excellent thermal and mechanical strength, tailorable structures, tunable ionic conductivities, smaller methanol permeabilities, and potentially lower costs. Past reviews on alternative PEMs have highlighted the different properties, modifications, and performances ofseveral types of aromatic-based membranes alongside Nafion and biopolymers. However, few recent reviews have focused solely on aromatic-based membranes. This paper aims to discuss the challenges associated with several known aromatic-based polymers in applications to LTPEMFCs, HTPEMFCs, and DMFCs, which include highlights of the latest studies on research related to their modifications, improvements, durabilities, and performances as protonicexchange membranes.

## 2. Types of Aromatic Polymer-Based PEMs: Properties and Development

The backbones of these polymers are linked together by aromatic and phenyl rings with C-C, C=C, and C-H bonds within their backbones that provide the membrane with excellent mechanical, thermal, and chemical strengths. These linkages include groups with varying numbers of ether and ketone functionalities, such as those in poly(ether ketone)s (PEKs) and poly(aryl ether ketone)s (PAEKs)-type polymers; sulfone functionalities in poly(ether sulfone)s (PESs), and polysulfones (PSFs); imide bonds in polyimides (PIs); benzimidazole rings in polybenzimidazole(PBI)-based polymers;ether-containing polyphenylene oxide (PPO), and more. Typical unit structures of the mentioned polymers are shown in Table 1. he ionic conducting properties of these membranes are ineffective in theirpristine form. To cater for their conductive properties, strong acidic, proton conductive sulfonic acid groups (-SO_3_H) are commonly introduced into these polymer chains through reactions with sulfonating agents, producing sulfonated derivatives of these polymers that are more suitable for application as PEMs. Other functionalities are also possible, depending on the final application, whether as PEMs for low- or high- temperature PEMFCs, or as AEMs. Examples of these other functionalities are quaternary ammonium, imidazolium, or benzimidazole groups [4,12,13,14,15,16]. 

Past research has highlighted that the strength of the proton conductivity of these membranes is governed by the concentration of effective ionic/proton conductive groups (referred to as the ion exchange capacity (IEC)), hydration levels, temperature, and hydrophilic/hydrophobic phase separation. Typically, within hydrated membranes, proton conduction occurs through the Grotthuss mechanism (proton hopping between ionic domains and water molecules) and vehicle mechanism (proton diffusion). In most cases for sulfonated polymers, the degree of sulfonation (DS) or IEC determines their hydrophilicity and conductivity, where a higher DS/IEC leads to a more hydrophilic membrane with better water uptake, hydration, and conductivities. However, a highly hydrophilic membrane tends to absorb water excessively. Weakened interactions between polymer chains through ionic and hydrogen bonds resulting from a large number of water molecules occupying the free volumes between chains can cause a large amount of swelling, mechanical deterioration, and other issues related to the oxidative stability and reactant permeabilities [15,17]. Furthermore, the smaller hydrophobic/hydrophilic phase separation of these polymers in comparison to Nafion leads to lowered methanol permeabilities, providing advantages in terms of the performance in methanol fuel cells [18,19]. However, researchers have also noted that the small phase separation contributes to low conductivities due to less connected ionic domains, meaning the formation of proton conducting channels is not as effective as in Nafion [20,21]. 

The mentioned advantages and disadvantages are shared bymost of the aromatic-based membranes considered in this review. However, other specific strengths and weaknesses may be displayed by individual polymers. Understanding the effects of the characteristic at a molecular level towards the function of the PEM as a whole candetermine their electrochemical performance, their durability under varying fuel cell conditions, and their lifetime. Past researchers have adopted strategies and modification techniques to design hydrocarbon-based PEMs with improved properties and performances. To utilize the advantage of high proton conductivities offered by these PEMs with high DS/IEC requires balance with their water uptake and mechanical strength, which remains a challenging task. Overthe years, several of the methods that have been appliedby researchers in an attempt to improve the properties of hydrocarbon-based PEMs include structural modifications (additional branching, pendant groups, etc.), crosslinking, polymer blending, mixed-matrix, block copolymerization, and the introduction of inorganic/organic fillers/nanofillers [5,7,22,23,24]. While these modifications yielded positive enhancements ofthe PEM, it is important to consider optimization between electrochemical properties; thermal, mechanical, and chemical strengths; water uptake; fuel resistance; and oxidative stability by carefully designing the polymeric structure andcontrolling the ratio of combination between materials. The potential to effectively utilize these PEMs in actual fuel cell conditions depends on how well their individual characteristics balance out.

### 2.1. Poly Aryl Ether Ketones (PAEKs)

Poly aryl ether ketones (PAEKs) refer to polymers consisting of different numbers of ether and ketone functionalities connecting aryl rings, such as polyether ketones (PEKs), polyether ether ketones (PEEKs), and polyether ketone ketones (PEKKs). More ether or ketone groups may be included and their positions within the chain depend on the monomers used at the beginning of PAEK synthesis. The chain may also contain alkyl groups or fluorinated functional groups. These polymers are semicrystalline thermoplastics withgood chemical and thermal stability, dielectric properties, and mechanical strength. Sulfonated poly(ether ether ketone) (SPEEK) is -SO_3_H-functionalized PEEK andis one of the most commonly studied PEMs for fuel cells. Its synthesis is normally carried out through the post-sulfonation of commercial PEEK with strong sulfuric acid (98% H_2_SO_4_). K. Kreuer [25] has proposed the difference between the microstructural arrangement of –SO_3_H groups in Nafion and the two-ketone SPEEKK, where there is less pronounced hydrophobic/hydrophilic phase separation in SPEEKK compared to Nafion. This leads to poorer connectivity between –SO_3_H ionic domains, and thus a smaller proton conductivity of SPEEKK. PEEK with cationic groups, such as quaternary ammonium and imidazolium, may be utilized as AEM [16]. 

Recent studies have focused on understanding the PEM properties of SPEEK membranes, including durability studies, which are important in predicting the membrane’s lifetime under fuel cell operating conditions [17,26,27,28]. Regarding the pure SPEEK, M. Parnian et al. [17] highlighted the changes in several key properties of SPEEK in their study of SPEEK with various DS values. The summary of some of their findings, stated in Table 2, revealed an increasing trend in water uptake, swelling, proton conductivity, and thermal degradation at an increasing DS, but deterioration in the tensile and oxidative stability. The oxidative stability refers to the membrane’s resistance to degradation due to peroxide radicals (HO• and HOO•) formed from incomplete oxygen reduction reactions at the fuel cell cathode. The SPEEK with the lowest %DS takes the longest time to completely disintegrate in Fenton’s reagent. With increasing %DS and higher water uptake, the radicals are more easily diffused into the membrane and attack the aromatic backbone, sothe membrane becomes rapidly disintegrated.

In terms of the processability and MEA performance, common organic solvents, namely DMSO, DMAc, DMF, and NMP, used for the solution casting of SPEEK also seemto influence the conductivity of high and low DS SPEEK. X. Liu et al. [29] found that low DS SPEEK casted from DMSO has a larger conductivity due to the weaker molecular interactions between residual solvent molecules and the polymer, while no significant change in properties wasobserved, regardless of the solvent type, for high DS SPEEK. The ex-situ mechanical degradation of SPEEK in hygrothermal cycle tests for 700 min conducted by S. H. Mirfasi et al. [27] showed permanent deformation and a 4 micron reduction of membrane thickness at the end of the tests, resulting in a faster hydrogen crossover rate. Under creep and tensile residual stress, the membrane wasdegraded due to fatigue and became more brittle, and the toughness dropped. Additionally, the degraded SPEEK exhibited an increase in water uptake and swelling-induced stress, thusworsening the dimensional stability. A. Karimi et al. [28] provided a study on the MEA model of SPEEK compared to Nafion MEA. The use of a SPEEK membrane in MEA at high temperatures is favorable when the pressure and water content at the anode gas feed arehigher, but the cell performance drops when the water activity is largely reduced at 140 °C. Additionally, the SPEEK proton conduction is smaller than that of Nafion. 

#### Improvements to SPEEK and Other PAEK-Based PEMs

Recently, researchers followed similar strategies to the modifications of SPEEK with the use of new or modified materials to improve their properties. Reaping the advantages of high DS SPEEK requires the intermolecular interactions between polymeric chains to bestrong enough to overcome swelling, especially at high temperatures. S. Gao et al. [30] investigated the properties of nanocomposite SPEEK with a high DS of 84% grafted with graphene oxide (GO-g-SPEEK), synthesized from the ‘grafting’ reaction between partially hydroxyl-functionalized SPEEK and brominated GO (GO-Br). The restraining effect of GO limited the SPEEK’s swelling, despite thetriple increment in water uptake compared to Nafion. Furthermore, its conductivity improved above 80 °C, which is the point where the proton conductivity of Nafion would drop due to dehydration. Blends of GO-g-SPEEK with Nafion-33 achieved a conductivity of around 0.22 S/cm at 90 °C, while its MEA performance reached a power density of 213 mW/cm^2^ compared to 112 mW/cm^2^ for unblended GO-g-SPEEK. This suggested that blending with Nafion provided better interfacial contact between the catalyst and membrane. Another study by S. Bano et al. [31] focused on crosslinked SPEEK with ethylene glycol (EG), followed by the incorporation of cellulose nanocrystals (CNCs). XRD analysis of the resulting XSPEEK-CNC showed the amorphous characteristic of the membranes. The hydrophilic sulfate and hydroxyl groups present on CNCs restore the loss in water uptake and IEC due to the crosslinking of SPEEK. An effective increment in proton conductivity is observed up to 4 wt% of CNC, attributed to the homogenous dispersion of CNCs, the presence of additional hydrophilic functionalities, interfacial hydrogen bonds, and good hydrophilic-hydrophobic phase separation. However, the XSPEEK-CNC is also a stiff membrane as a result of crosslinking.

**Figure 1:**
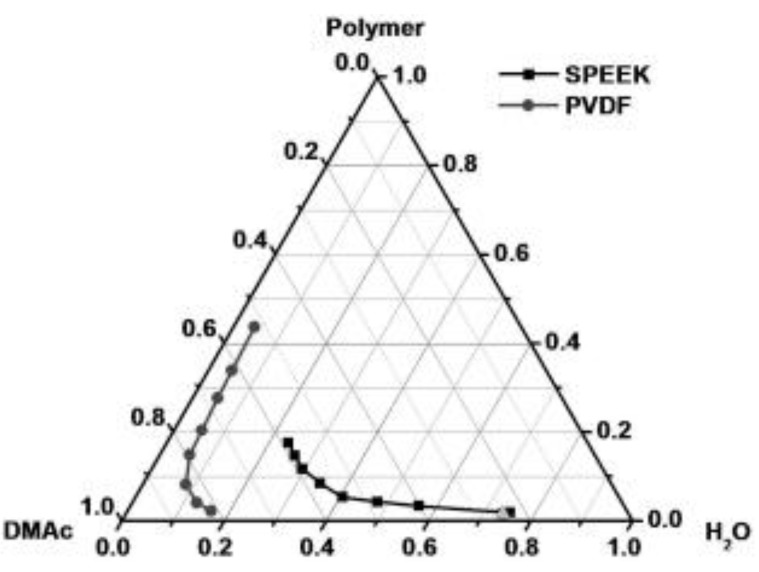
Cloud point curves of SPEEK/dimethylacetamide (DMAc)/H_2_O (square plot) and poly(vinylidene fluoride) (PVDF)/DMAc/H_2_O (circle plot) [32].

**Figure 2:**
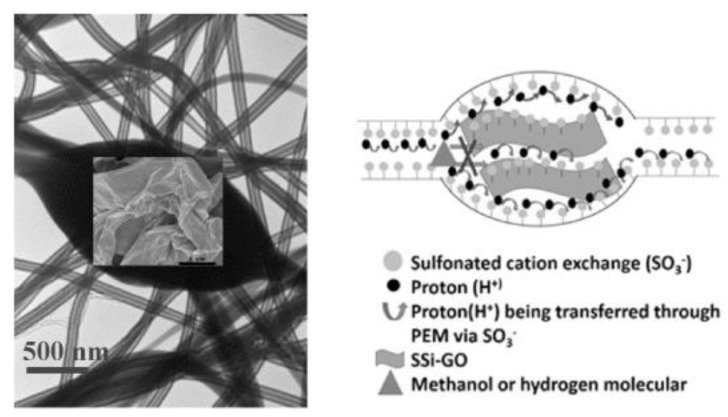
TEM image of a co-spinning sulfonated organosilane graphene oxide (SSi-GO)/SPEEK and aschematic representation of methanol and hydrogen diffusion through the cambiform-like structure of the core-shell nanofibers [33].

Morphological modifications through the introduction of nanofibers also showed several advantages. G. Liu et al. [32] studied the properties of SPEEK-impregnated poly(vinylidene fluoride) (PVDF) electrospun nanofibers for DMFC. SPEEK (76.7% DS) wasembedded in PVDF nanofibers through a cloud point polymer/solvent/non-solvent (SPEEK/DMAc/H_2_O) ternary system (Figure 1) to prevent PVDF nanofibers from collapsing when mixed with SPEEK (as PVDF is also soluble in DMAc).The SPEEK-embedded PVDF nanofiber membrane wasfound to be more flexible with a sustained yield and modulus. Its methanol permeability washalf of that of pristine SPEEK and 1/3 of that of Nafion 115. This advantageous property led to a higher peak power density at 104 mW/cm^2^ compared to Nafion 115 at 84 mW/cm^2^ in DMFC MEA that utilized 5M methanol. Y. Wu et al. [33] fabricated SPEEK core-shell nanofibers containing sulfonated organosilane graphene oxide (SSi-GO) nanosheets through coaxial electrospinning, forming a cambiform-like morphology in the membrane. The co-spinning SSi-GO/SPEEK exhibited higher water uptake than the membranes formed from casting and mono-spinning, where wrinkled voids in SSi-GO weresuggested to act as a water reservoir, while the SSi-GO restricted swelling. The methanol permeability was 39% of that of Nafion 115, despite being thinner. The proton conductivity of the membrane containing 2.5 wt% SSi-GO was1.24 and 1.42 times higher than that of mono-spinning and casted membranes, respectively, likely due to the uniform dispersion of SSi-GO, axial arrangement of SSi-GO with SPEEK nanofibers to form cambiform core-shell nanofibers (Figure 2), higher water uptake, and conductive sulfonic and hydroxyl groups. 

In terms of other PAEK membranes, K. Kang et al. [34] investigated the properties of a semi-interpenetrating network (IPN) consisting of a multiblock copolymer of PAEK-b-KSPAEK with organosiloxane (OSPN). The OSPN-containing membrane displayed an increased elasticity and reduced permeation of oxidative radicals and methanol. Because of the hydrophilic characteristics of OSPN, which elevated the PAEK-b-KSPAEK’s water absorption, the proton conductivity achieved was close to that ofNafion 115. PAEK polymers have also been utilized in HTPEMFC. J. Li et al. [35] characterized the properties of a PA-doped brominated tetramethyl PAEK (BrPAEK) decorated with four types of nitrogen-heterocyclic molecules (Pyridine, 1-methylimidazole, 1H-benzotriazole, and 3-amino-1,2,4-triazole). Pyridine- and 1-methylimidazole-containing BrPAEK were the only membranes showing a PA absorption ability, andthe latter contributed to the BrPAEK with the highest conductivity at 170 °C, in ananhydrous state. The conductivity exceeded that of PBI when there was asufficient content of imidazole per unit, in which a larger PA doping level waspossible. However, more imidazole per unit negatively affects the oxidaticve stability. Furthermore, J. Yang et al. [36] studied the effects of blending PVDF and PVDF-HP (poly(vinylidene fluoride-co-hexafluoride) with 1-methylimidazole-containing BrPAEK (MeIm-PAEK). The presence of PVDF and PVDF-HFP stabilized the membrane by reducing the PA uptake and swelling, as well as enhancing the tensile strength, as the pristine MeIm-PAEK was unstable due to the high bromination degree (45%). Blends with PVDF-HP exhibited better PA uptake and proton conductivity, yet a smaller mechanical strength, which was likely due to the presence of large trifluoromethyl groups. 

Table 3 summarizes some of the recent literature on the improvements to PAEK and PEEK membranes. Focus is given tothe proton conductivity, as this influences the PEM performance, along with IEC and water uptake, which affects proton transport. The electrochemical aspects do not show exhibitdifferences between PAEK and PEEK, and upon functionalization to SPEEK and SPAEK or other functionalities, they show a similar dependency to DS, IEC, water uptake, and PA uptake (HT-PEMFC application). Their thermal strengths arewithin the range of fuel cell operations below 200°C. Furthermore, their mechanical stabilities also depend on the aforementioned factors, where modifications that enhance the strong intermolecular interactions between polymer chains, such as crosslinking and the use of fillers, can allow the utilization of SPEEK or SPAEK with a higher DS. It is noted that more literature seems to be available for SPEEK compared to other SPAEK- or PAEK-type polymers. SPEEK’s synthesis through the post-sulfonation of commercially available PEEK is likely simpler and the DS is easier to control, while more complex polymerization reactions may be required to prepare other PAEK-type polymers with differing structures. 

### 2.2. Polyimide (PI)

The formation of charge transfer interactions between the dianhydride and diamine functionalities of the polyimide (PI) backbone provides the polymer withexcellent thermal stability. PI membranes also exhibithighmechanical strength and chemical stability. The structure and properties are tailorable by using different dianhydride and diamine monomers. Sulfonic acid-containing sulfonated polyimides (SPIs) synthesized by using sulfonated precursors, such as 4,4’-diaminodiphenyl ether-2,2’-disulfonic acid (ODADS), 4,4-diaminostilbene-2,2-disulfonic acid (DSDSA), and 4,4’-Diamino-2,2’-biphenyldisulfonic (DAPS), result in PI membranes with proton conductive properties [12,43,44] Final structures of PIs are tailorable and block copolymers may also be formed.Aside from thechallenges related to ahigh DS and IEC, it has also been highlighted that the imide rings are very sensitive to water, sothe unit is prone to hydrolysis, negatively impacting the hydrolytic stability [45].

#### Improvements to SPI PEMs

In recent years, several studies have investigated the potential of SPIs with varying molecular structures, while polymer blending and filler additives have also been attempted. Z. Yao et al. [46] aimed to improve the hydrolytic stability of SPIs by synthesizing perylenediimine-containing, aliphatic-type SPIs with different chain lengths, through the mild polyacylation of a sulfonated diarene monomer and aliphatic perylenediimide dicarboxylic acid monomers. Both short and long aliphatic SPIs showedimproved hydrolytic stability, compared to normal SPIs, under tests in hot water at 80 °C for 300 h, where the likely factors included the rigid perylene structures that helped strengthen the polymer. However, the long-chain AL-SPI-10 became a bit fragile after the test. Furthermore, shorter-chain AL-SPI-5exhibited better IEC and water uptake than the long-chain counterpart, while its proton conductivity was higher than that ofNafion 115 at 80 °C. K. Liaqat et al. [47] characterized a novel sulfonated polyimide (NSPI) with a unique structure inwhich the –SO_3_H groups wereattached to a phenylene side chain rather than the main chain, via the copolymerization of novel sulfonated diamine (NSDA) with 4,4’-oxydianiline (ODA) and 4,4’-oxydiphthalic anhydride (ODPA). The relocation of –SO_3_H groups to the side chain was suggested to improve the hydrolytic stability of NSPI under the test at 140 °C with pressurized steam, by lowering the risk of the nucleophilic attack of hydroxyl ions on the imide rings. The power density of the NSPI with a 10/90 ratio of NSDA/ODA exhibited a slightly elevated peak power density of 28.7 mW/cm^2^ compared to Nafion 117 (25 mW/cm^2^) in DMFC MEA at 70 °C. These reports highlight that a better hydrolytic and oxidative stability of SPI ispossible if there is a distinct separation between the –SO_3_H groups andthe imide rings.

S. Feng et al. [48] applied the charge transfer (CT) complex method to prepare CT complex blend membranes using electron-accepting SPIs and electron-donating polyethers (PEs). The SPI/PE CT complexes were subjected to heat treatment. While the heat-treated membranes displayed improvements in thetensile strength, elongation at break (EB), and Young’s modulus compared to non-heat-treated membranes and normal SPI, other properties were characterized for non-heat-treated membranes due to issues with heat treatment and reproducibility. The thin CT complex membranes with a thickness from 13 to 28 microns displayed hydrogen permeabilities that were 4.1 and 5.4 times smaller than those of Nafion 212, which wasconsidered as an advantageous characteristic for thin membranes. Despite the smaller values of IEC, proton conductivity, and peak power density, the thin SPI/PE was found to be stable under the 10 h MEA test. Nanofibers based on different structures of SPIs were investigated by G. Ito et al. [49]. Block-types SPIs and graft-type SPIs both exhibited the properties of self-assembling hydrophilic/hydrophobic microphase structures in a membrane state. It was found that the fluorine-concentrated SPI nanofibers hadlower proton conductivities that were attributed to their fragility when dehydrated. On the other hand, less-fluorinated sulfonated random polyimide (S-r-PI) nanofibers achieved mechanically stable, low gas permeation, and high proton conductive characteristics. Hence, the S-r-PI nanofibers were integrated into a sulfonated block-graft polyimide (S-bg-PI) polymer matrix to form a Nanofiber Framework (NfF) composite membrane with a 12 micron thickness. The nanofibers reinforced the membrane mechanically and improved the thin membrane’s gas permeability. Furthermore, the composite’s proton conductivity was comparable to and even exceeded that, of Nafion NR212 at temperature range between 30 to 90 °C and 95% RH. 

Aside from structural modifications, filler addition to the SPI matrix has also shownseveral improvements. Recently, P. Y. You et al. [50] incorporated rice husk ash (RHA) biofillers into the SPI matrix. A suitable concentration of RHA resulted in stiffer membranes and higher water uptake than normal SPIs. At a 15 wt% RHA content, the proton conductivity was found to be double that of Nafion. This improvement canlikely be attributed tothe Lewis acid-base interaction between SPI chains, and the hydroxyl groups of RHA, which attract water molecules (high water retention). However, it should be expected that alarge amount of RHA can block the continuous proton transfer channels.

Some of the recent studies onSPI membranes are summarized in Table 4. DS is not normally specified for SPI, yet the trend of water uptake and conductivity still follows that of IEC, as reported. SPI membranes are physically stable enough, even under conditions of HTPEMFC, providing that the PEM’s water or PA retention, mechanical strengths, and hydrolytic stabilities are controlled. 

### 2.3. Polyether Sulfones (PESs) and Polysulfones (PSFs)

Polysulfones (PSFs or PSUs) are polymers consisting of sulfone and ether linkages. PSF refers to apolymer that also contains an alkyl group, while the shorter chain polyethersulfone (PES) refers to apolymer chain with only sulfone and ether groups. Similar to PAEK and PI, PSF and PES polymers possess high mechanical, thermal, and chemical strengths. PES is denser, with a higher glass transition temperature (~220 °C) compared to PSF (~185 °C) [52]. A number of research studies are available for AEMs based on PSFs, although PSF membranes are also utilized as PEMs. The synthesis of proton conducting sulfonated polyether sulfone (SPES) may be done through using precursors such as 4,4’-Dichlorodiphenylsulfone (DCDPS), or the sulfonation of commercial SPES [53,54]. Related durability issues, particularly theloss in mechanical strength upon a higher degree of sulfonation, limit this polymer’s long-term lifetime under an actual fuel cell environment, requiring necessary modification strategies to tailor the morphological and molecular aspects. 

#### Improvements to PES- and PSF-type PEMs

Improvements inPSF and PES membranes are also focused on increasing theirconductivity and durability. S. Matsushita and J. D. Kim [55] attempted the thermal crosslinking of sulfonated poly(phenylsulfone) (SPPSU) with ethylene glycol and glycerol crosslinkers using water as a solvent, with annealing temperatures ranging from 80 to 200 °C. SPPSU prepared with 6 mol/rpu ethylene glycol at 200 °Cwas the membrane with optimized dimensional properties, with an acceptable proton conductivity, despite being lower than 0.1 S/cm. Nevertheless, this study suggested the possibility of using water instead of organic solvents in membrane preparation, as well as the importance of controlling the membrane’s physical stability, as there are concerns that it may be damaged during MEA preparation. N. Urena et al. [56] attempted the “one-pot two-step synthesis” approach to synthesize high molecular weight, multiblock copolymers composed of PSU and PPSU using commercially available monomers, followed by sulfonation using trimethylsilyl chlorosulfate. This approach is suggested to be less complex than the polycondensation of monomers forproducing high molecular weight polymers, which may be better for industrial-scale processes. The obtained sulfonated multiblock copolymers (SPSU/SPPSU) had no obvious phase separation but achieved a higher hydration level than Nafion 117. The tensile strengths of the dry membranes exceeded that of Nafion 112, which increased with IEC. Tensile drop was more prominent for the high IEC membrane in a wet state, but still considered as acceptable (55 MPa for the membrane with 1.58 meq/g IEC). The maximum power density of the multiblock SPSU/SPPSU was ~400 mW/cm^2^, which was in between Nafion 112 (729 mW/cm^2^) and Nafion 117 (310 mW/cm^2^) at 70 °C and 100% RH. 

S. Gahlot et al. [57] studied the effects of sulfonated mesoporous silica (S-MCM-41) on SPES. Similar to most inorganic-organic composites with porous, hygroscopic fillers, the presence of S-MCM-41 in the SPES matrix increased thewater uptake and tensile strength. The content of bound water was 0.51% for the filler-containing SPES and 0.2% for normal SPES, showing the filler’s ability in water retention. While IEC showed an increasing trend forthe S-MCM-41 content in SPES, the highest proton conductivity was achieved with 2 wt% S-MCM-4, due to the uniform filler distribution, higher porosity, and proper ion channel formation. X. Xu et al. [58] prepared cellulose whiskers (CW) functionalized with various Fmoc-amino acids (Glycine, 5-amino-Valeric acid, ʟ-serine, ʟ-Aspargine, and ʟ-Leucine). The Fmoc-protecting groups were removed and incorporated into the SPSF matrix to form a proton conductive mixed-matrix membrane. SPSF containing 10wt% of ʟ-serine-functionalized CW achieved the highest conductivity at 0.234 S/cm at 80 °C. The highly hydrophilic functionalized CW and presence of –NH_2_ from amino acids provided the membrane with more water and conductive groups. Moreover, the methanol resistance was improved. The power density of SPSF/CW-Ser projected a peak power density of73.757 mW/cm^2^, whereas for Nafion 117, thevalue was 51.323 mW/cm^2^, at 60 °C and 100% RH in single-cell DMFC MEA with 2M methanol.

**Figure 3:**
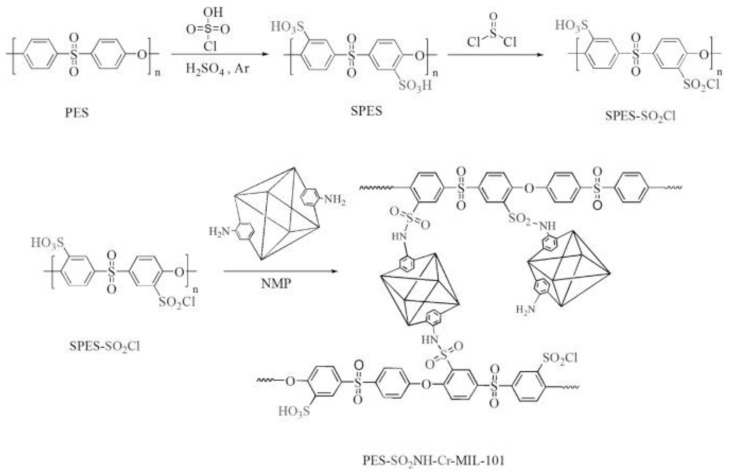
Schematic of the procedure for the synthesis of PES-SO_2_Cl with Cr-MIL-101_2_metal organic framework (MOF), utilized as PA-doped PEM for HTPEMFC [59].

A few recent studies have also characterized modified PES-type polymers as PEMs for HTPEMFCs. N. Anahidzade et al. [59] utilized an amino-functionalized metal organic framework (MOF) (Cr-MIL-101-NH_2_) on chlorosulfonatedpoly(ether sulfone) (PES-SO_2_Cl), resulting in crosslinking (Figure 3). While the PA uptake was less, the PA retention, proton conductivity, and thermal and mechanical strengths werebetter for the MOF-containing membrane. The conductivity achieved was 0.041 S/cm at 160°C, in an anhydrous state. Moreover, a 14 day durability test under the same conditions also highlighted a less significant drop in conductivity within two days, before remaining stable until the end of test, showing the advantage of the acid-stable, porous MOF towards PEM stability. H. Bai et al. [60] introduced graphitic carbon nitride (CN) nanosheets into a poly(ether sulfone)-poly(vinyl pyrrolidone) (PES-PVP) matrix. CN nanosheets raised the PA uptake and proton conductivity of the composites to a 0.5 wt% nanosheet content. The proton conductivity increased by 36% for the composite with 0.5 wt% CN, compared to the pure PES-PVP at 160°C, in an anhydrous state. The CN nanosheets weresuggested to interact with PA molecules and improve the rate of ionization of free PA and protons from PVP.

Table 5 provides asummary ofseveral improvements to the sulfone group-containing PES, PSF, and phenyl sulfone (PSU). There are similarities inthe performance of sulfone-containing PEMs when compared to that of ketone-containing PEMs, where DS and IEC affect the water uptake and conductivity. It is also worth noting that the ketone and sulfone groups can be combined into one polymeric chain, which has beenreported in several studies. An example is the recent study conductedby J. Xu et al. [61] on crosslinked sulfonated poly(aryl ether ketone sulfone) (C-SPAEKS) with multiple sulfonic acid side chains. The existence of interaction by crosslinking restricted swelling and controlled the methanol permeability, whereas the IEC being larger than Nafion 117 offered better conductivities. Despite the positive outcome forkey properties, the power density was lower than that of Nafion 117, which may be due to the compatibility of the membrane within the components in the MEA. 

### 2.4. Polybenzimidazole (PBI)

Mechanically and thermally strong polybenzimidazole (PBI) is of interest foruse as a PEM in HTPEMFC that operates in the temperature range of120 to 180 °C. Unlike PAEK-, PI-, or PES-type polymers that require additional functionalization to allow binding to PA molecules, the benzimidazole rings that naturally exist in the PBI backbone play this important role, enabling proton conduction to take place via both the Grotthuss and vehicular mechanism, between PA molecules (free and bounded to the –NH of benzimidazoles) and water molecules. The key properties, including the acid doping level (ADL) (or percentage PA uptake (%PA)), mechanical and thermal strengths, and proton conductivity, change with respective PBI types. The proton conductivity of PA-doped PBI membranes depends on the ADL, where more acid retained in the membrane leads toincreased conductivity. At a high ADL, the membrane’s tensile strength is lowered due to the plasticizing effect of PA, despite the better conductivity achieved. Furthermore, as PA remains in liquid-form embedded within the membrane’s free volume, leaching occurs over its lifetime of usage. In the MEA, PA leakage appearsto be more prominent on the cathode side of the membrane; in which water vapor produced from the cathode reaction canfacilitate the removal of PA. The leakage rate increases at a higher current density. Acid loss subsequently leads tohigher cell resistance and conductivity drop [66]. It is worth mentioning that several types of PBI, namely meta-PBI and ABPBI, dissolve poorly in common organic solvents, such as dimethylacetamide (DMAc), dimethylformamide (DMF), and N-methyl-2-pyrollidone (NMP), affecting the processability and membrane formation, due to their rigid structure and high glass transition temperature [67]. However, alternative acidic solvents may be utilized. N. Ratikanta et al. [68] studied the effect of methane sulfonic acid (MSA), trifluoroacetic acid (TFA), formic acid (FA), and sulfuric acid (SA) as solvents for ABPBI. The polymer casted from TFA displayed the highest PA absorption and proton conductivity at 150 °C, but had the weakest mechanical strength. The MSA-casted membrane offered the best cell performance with a small electrolyte resistance. 

#### Improvements to PBI-Type PEMs

Under HTPEMFC conditions, the PEM is subjected to a faster rate of thermal and chemical degradation, mechanical stress, and PA loss. Researchers have followed similar strategies to improve the properties of PA-doped PBI similar to that of other hydrocarbon-based ion exchange membranes, aiming to balance the acid uptake and proton conductivity with their physical properties, as well asovercome the solubility issue and slow down the acid leaching rate. Aside from meta (m)- or para (p)-PBI and short-chained ABPBI, several PBI-based polymers with varying backbone structures have been synthesized in past studies. By the polymerization of different monomers, sulfonated PBI (SPBI), pyridine PBI (Py-PBI), PBI with ether bonds (OPBI), fluorinated PBI (F6-PBI), and branched PBI each have specific properties. Some have exhibited a better solubility, PA uptake, mechanical properties, and proton conductivity compared to m-PBIs [67,69]. They may also be modified further by crosslinking, introducing additional side-chains, blending, filler additives, and so on. Recently, the effects of new materials and modification techniques have been investigated. 

**Figure 4:**
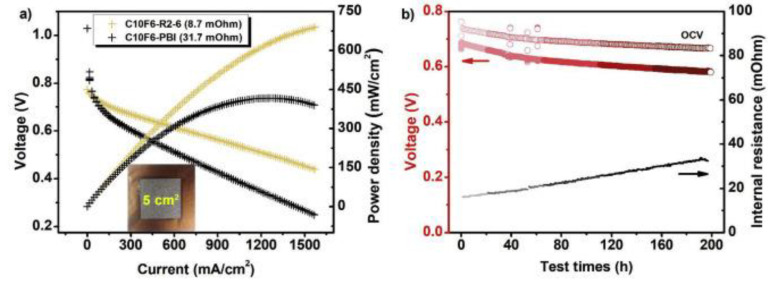
Outcomes of the crosslinked fluorinated PBI (F6-PBI) with unrestricted –NH sites (**a**) and the change in OCV of the membrane during a 200 h durability test, under 160°C, and in an anhydrous state (**b**) [73].

X. Li et al. [70] grafted additional benzimidazole groups onto the backbone of aryl-ether PBI (Ph-PBI) through an N-substituted reaction without catalysts. The polymer solubilities of Ph-PBI and grafted Ph-PBI wereexcellent for most of the common solvents. The ADL and proton conductivity increased with a highergrafting degree. At 200 °C, the conductivity of the Ph-PBI with the maximum grafting degree reached 0.235 S/cm. However, the tensile stress wasparticularly low for the membrane due to the high ADL (3.2 MPa, slightly higher than OPBI, which was 2.5 MPa). For operation under the temperature concerned, it is important to consider how stable the membrane would be in the long term. H. Chen et al. [71] proposed a concept of dual proton transfer from a crosslinked membrane consisting of PBI with proton-donating and -accepting properties with polymeric ionic liquid (PIL) based on BuI-PBI and anion BF^-^ as proton acceptors. The anionic part of PIL wasable to accept protons to enhance the PA capacity, at the same time accepting protons through electrostatic interaction. The anions of PIL facilitated proton transfer; therefore, the conductivity increased. In contrast, the PIL caused a slight reduction in Young’s modulus and the ultimate tensile strength (undoped), likely due to the destruction of hydrogen bonds in PBI during the N-quaternization reaction, forming PIL. With the inclusion of crosslinking, some –NH sites were important for PA absorption and proton transfer. L. Wang et al. [72] attempted tospare the –NH sites from the crosslinking reaction by the synthesis of branched F6-PBI with bis(3-phenyl-3,4-dihydro-2H-1,3-benzoxazinyl) isopropene (BA-a) as the polymeric covalent crosslinker. The unrestricted –NH sites, branched structure, and amine groups of crosslinkers helped in PA absorption and retention. The stability wasmaintained as a more rigid membrane was produced as a result of crosslinking; beneficial for the membrane in terms of itretaining its strength in a doped state and at high temperatures. Moreover, the membrane achieved a 690 mW/cm^2^ peak power density at 160°C in H_2_/O_2_ HTPEMFC MEA tests. Long-term stability tests for 200 h also recorded stable open circuit voltage (OCV)(Figure 4), owing to the low H_2_ permeability and internal resistance, high stability, and acceptable oxidative stability. 

Conducting multiblock copolymerization to produce strong, phase-separated membranes is common among aryl ether ketone- and sulfone-type polymers. Recent attempts have been made to produce PBI-type block copolymers. L. Wang et al. [74] synthesized block copolymers consisting of varying ratios of OPBI and p-PBI segments. The resulting copolymer showed a nanophase-separated morphology that provided a larger free volume; hence, at equal ratios of OPBI and p-PBI, the ADL value was7.9 PA/rpu, which was higher than that of the individual segments (5.8 PA/rpu for OPBI and 4.7 PA/rpu for p-PBI). Furthermore, the equal ratios in the block copolymers exhibited the maximum phase separation degree. Therefore, there was alarge continuous channel for proton transfer. In turn, the proton conductivity achieved is stated to be five times higher than that of the individual segment. The peak power density reached 360 mW/cm^2^, which is an obvious improvement (p-PBI: 250 mW/cm^2^ and OPBI: 268 mW/cm^2^) under H_2_/air at 160°C, in an anhydrous state. Although there are advantages on the electrochemical side, the phase separation in a copolymer’s microstructure may cause the membrane to be more susceptible to cracks because of the presence of nanocracks. This will make the membrane more likely to rupture under continuous stress.

Compatible fillers like silica, metal oxides, and graphene oxide are still utilized to enhance PBI properties. Functionalized fillers potentially provide stronger intermolecular interactions to minimize swelling due to a high PA uptake, while maintaining the conductivity. E. Abouzari-Lotf et al. [73] have shownthe enhancement of PA retention and proton conductivity of 2,6-Py-PBI phosphonic acid-functionalized graphene oxide (PGO). The PGO appeared to reduce the crystallinity of the polymer matrix while being able to maintain a sufficient mechanical strength. The more amorphous structure, which, according to the authors, provided stronger sites for PA retention and proton hopping pathways, increased the conductivity of the composite. The conductivity was also observed to increase with a small level of humidity (10% RH), where it was thought that the humidity reduced the contact resistance between the gas diffusion layer (GDL) and the membrane. A slower drop in conductivity was observed for the composites with PGO, shown in a 20 h durability test at 140 °C, without hydration, indicating a potentially stable membrane.

Table 6 provides asummary ofPBI-type membranes. Even recent studies have highlighted the advantages of PA-doped PBI membranes as solid electrolytes for HTPEMFCs that operate above the boiling point of water. At an appropriate ADL, it is feasible for PBIs to reach conductivities similar to those of Nafion in a temperature range of 160–200°C. Similarly, for the case of water-retaining SPEEK, SPI, and SPES, strengthening the PBI molecular chain interactions to minimize swelling and the risk of fast disintegration at a large ADL and high temperatures is necessary, especially when a high proton conductivity is desired. On the other hand, effective single-cell performance of the membranes would also rely on the PBI’s compatibility with the MEA components, gas permeability, oxidative stability, and degradation rate over its lifetime. 

### 2.5. Polyphenylene Oxide (PPO)

Polyphenylene oxides (PPOs), also referred to as polyphenylene ethers (PPEs), are ether-containing aromatic polymerswithelectrical insulation properties, high mechanical strengths, and good chemical resistance. In terms of their thermal aspects, the glass transition temperatures of PPO may reach as high as 210°C, although this varies, depending on their grade and modifications. The excellent dimensional stability of PPO is related to itslow moisture absorption, even when exposed to boiling water [83]. Modified PPO or PPE-based AEMs for fuel cells have beenwell-studied in past years; however, several recent studies have also reported the potential use of modified PPOs as PEMs. 

#### Improvements to PPO-Type PEMs

Similar to other sulfonated aromatic PEMs, SPPO’s IEC, water uptake, and conductivity relieson its degree of sulfonation. However, the weaker thermal stability and mechanical strength of the SPPO membrane may shorten its lifetime. I. Petreanu et al. [84] incorporated silica particles into the SPPO matrix and reported a higher ultimate tensile strength for the silica-containing membrane in a hydrated state, compared to pristine SPPO. Other key properties, including IEC and water uptake, lowered in the presence of silica. Beside SPPOs, the bromomethylated PPOs (BPPOs) with hydrophobic properties caneffectively improve the methanol resistance and DMFC cell performance of SPEEK, as investigated by X. Liu et al. [85], who employed a SPEEK-BPPO blend membrane. While the water uptake and conductivity wereaffected in the presence of BPPO, the excellent methanol resistance and selectivity contributed to the better DMFC single-cell performance at a 5M methanol concentration, achieving a power density five times higher than that of Nafion 117.

Recently, there havealso been attempts toutilize PPOs atoperating temperatures above 100 °C. X. Zhu et al. [86] prepared a crosslinked methylimidazole-functionalized PPO incorporated with phosphonic acid-functionalized siloxane as a PEM for high-temperature and low-humidity conditions. The effective proton conductive properties of the functionalized siloxane allowed the conductivity to further elevate after 100 °C at 5% RH, while crosslinking strengthened the membrane’s mechanical properties and oxidative stability. This suggests that a crosslinker functionalized with effective proton conductive functional groups has a positive role in simultaneously enforcing the membrane and improving the electrochemical properties. Furthermore, an investigation of a crosslinked PPO containing a triazole side chain, synthesized by J. Jang et al. [87], showed that the highly crosslinked structure wasable to minimize PA leaching, whilstimproving the mechanical and thermal strength, but also suppressed high PA absorption, leading to lower conductivities. The addition of more triazole side chains raises the PA uptake. Since the PA uptake still has a significant effect on theproton conductivity, the membrane must retain a suitable amount of PA for better conductivities. Therefore, this would require the optimization of both the degree of crosslinking and triazole content to balance out the key aspects to function as effective PEM. 

Table 7 summarizes several of the recently investigated PPO-type PEMs. While the number of studies for PPO-type fuel cell PEMs seems limited compared to AEMs, there is still good potential for the utilization of PPO as membrane material. Much like the other aromatic-based membranes, the optimization of individual aspects of the membrane is an important step in achieving electrochemical and durability balance.

## 3. Challenges and Future Perspectives

As seen from the extensive research conductedin past and recent years, PEMs derived from various hydrocarbon-based polymers hold a lot of potentials to be applied as solid electrolyte alternatives to Nafion for LTPEMFCs, HTPEMFCs, and DMFCs. The different modification methods employed to enhance these PEMs have observed that improvements intheir electrochemical characteristics and durability are possible, taking into account thecontrol, adjustment, and optimization of individual key properties. While several studies have recorded the performance of hydrocarbon-based membranes and their modified forms in long-term MEA tests, knowledge on characteristic changes in long-term durability under fluctuating temperatures, pressures, and fuel flows in actual fuel cell stacks could be more comprehensive. 

Nafion has been widely commercialized as it has a role as the standard in fuel cell systems and their production has been achieved at anindustrial scale. PEEK, PES, PI, PBI, and PPO polymers are already manufactured atlarge scales to cater for their diverse applications besides PEMs. However, suitable proton or ion conducting PEEK, PES, PI, PBI, and PPO specifically for fuel cell applications have yet to enter the competitive market alongside Nafion. The difficulty incommercializing these alternative PEMs is due to the durability issue and stability related to their electrochemical properties, which still requires further optimization. Hydrocarbon-based PEMs synthesized from individual monomers may hold some advantages in long-term durability compared to those derived from commercial polymers. However, the polymerization reaction process can be complex, andthe stoichiometric ratios, reaction conditions, purification, recovery, and film formation process must be specified at an upscale level, sothe economic feasibility needs consideration. Using commercial polymers that undergo functionalization reactions, blending, or filler inclusion may be a more viable option. Again, the functionalization level (ex: DS), MEA compatibility, and durability should be optimized. The cost of secondary materials (blended polymer, fillers, etc.) also needs to be included. 

## 4. Conclusions

In summary, aromatic-based membranes consisting of strong aryl rings, ether, ketones, sulfones, imides, and benzimidazole linkages, along with the optional inclusion of fluorinated structures, followed by functionalization with strong ionic conducting groups, provide the membrane with the mechanical, thermal, and chemical strengths; water or PA retention abilities; and ionic/protonic conductive properties required for them to function as PEMs for fuel cells operating in the temperature range fromambient to around 200°C. Furthermore, the low methanol and gas permeation of these alternative PEMs even offer benefits toward better MEA performances. Larger IEC and water absorption of high DS SPEEK, SPES, and SPI would lead to higher proton conductivities, that would otherwise be lower than those of Nafion due to smaller hydrophobic/hydrophilic phase separation and the less effective ion transport channel of hydrocarbon-based PEMs. On the other hand, high PA uptake or ADL- of PBI-type membranes, as well as imidazole or ionic liquid-functionalized PAEK, PES, Pi, or PPO, facilitate conductivity under anhydrous conditions. However, the excessive accumulation of water or PA molecules within the free volumes of these polymers can cause large swelling, deterioration in the mechanical strength, easier fuel permeation, and faster degradation that may becaused bypoor oxidative stability, which is adisadvantage in terms ofthe hydrocarbon-based PEM’s durability, thus affecting their lifetime under fuel cell operating conditions. 

To this day, there have been various strategies and methods adopted by researchers to enhance the PEM characteristics of aromatic-based polymers. Crosslinking, multiblock-copolymerization, the introduction of inorganic/organic fillers/nanofillers, the addition of branching or pendant structures, and morphological modifications through the inclusion of nanofibers within the polymer matrix have beenproven to improve the mechanical, thermal, oxidative stability, fuel resistance, and electrochemical performance of these alternative PEMs, especially concerning the utilization of the benefits of PEMs with a high DS and water/PA uptake. However, some challenges still remain for these PEMs, even as they have been further modified. Crosslinking could lead to brittleness. Filler/nanofiller addition may form more tortuous proton conductive pathways. The compatibility of these modified aromatic-based PEMs forthe MEA components may be different fromthat of Nafion and more elaborate investigations are needed, such as explorations on the electrolyte-electrode contact, catalyst quantity, and flowrates of reactants and oxidants. Nevertheless, aromatic-based PEMs still hold great potential as effective and low-cost alternative PEMs for fuel cells. The success of producing PEMs with excellent performances dependson the balance between the electrochemical properties, physical characteristics, MEA compatibility, and durability, which requires a careful in-depth understanding of the fundamental characteristics of the polymers and the optimization of individual aspects of the membrane. 

## Figures and Tables

**Table 1:** Typical unit structures of several aromatic-based polymers used as proton exchange membrane (PEM) materials.

Polymer Name	Typical Unit Structures
Poly aryl ether ketone (PAEK)	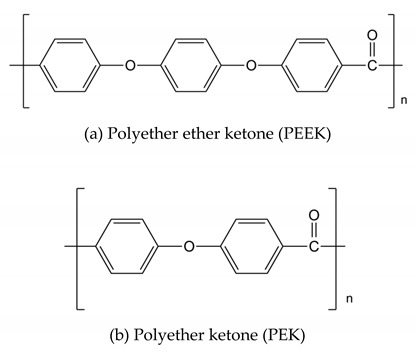
Polyimide (PI)	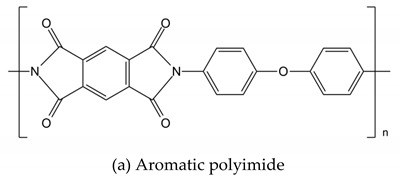
Polysulfone (PSF or PSU)	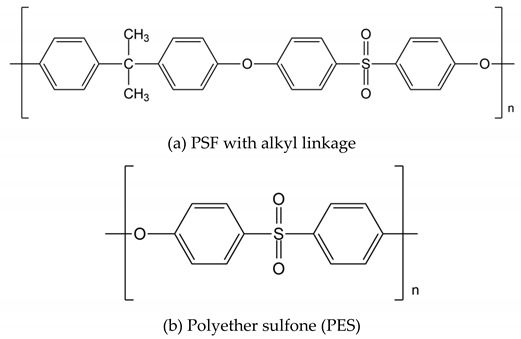
Polybenzimidazole (PBI)	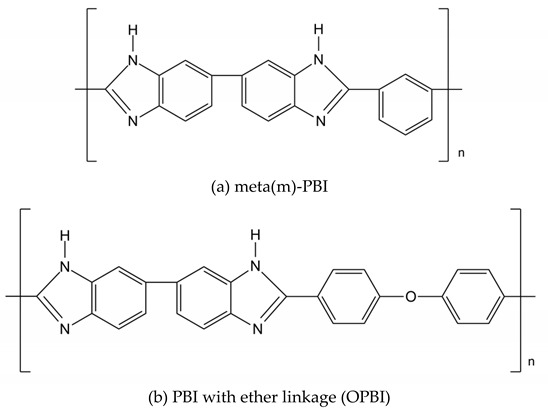
Polyphenylene oxide (PPO)	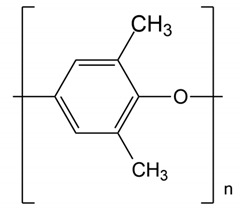

**Table 2:** Property changes in the sulfonated poly(ether ether ketone) (SPEEK) membrane with varying degrees of sulfonation [17].

Degree of Sulfonation (%)	Water Uptake (%, RT)	Thickness Swelling Ratio (%, RT)	Proton Conductivity (S/cm, 80 °C)	Tensile Strength (MPa)	Thermal Stability (% Degradation to 600 °C)	Oxidative Stability (~min)
40.23	6.29	2.13	0.2571	73	44	200
65.52	14.62	12.44	0.3003	63	46	56
75.95	52.01	27.20	0.4252	50.45	50	<6
89.23	97.98	34.54	0.4649	41	56	<2

**Table 3:** Water uptake and electrochemical properties of PAEK-type PEMs.

PAEK-Type									
Membrane	Year	%DS	Modifications	Fuel Cell Type	Filler Content	IEC (meq/g)	Water Uptake (%)	Proton Conductivity (S/cm)	Peak Power Density (mW/cm^2^)
SPEEK/n-BuOH [37]	2015	-	n-BuOH self-organization inducer	PEMFC	-	1.5 (mmol/g)	~52 (80°C)	0.314 (80°C)	-
b-CPAEK [38]	2016	-	PAEK block copolymers	DMFC	-	1.92	~50 (90°C)	~0.11 (90°C, 95%RH)	-
SPEEK/AIT [39]	2016	68	Amine-functionalized iron titanate (AIT)	PEMFC	2wt% AIT	-	72 (25°C)	0.12 (80°C)	204 (80°C, 90%RH, H_2_/O_2_)
Pore filling SPAEK [40]	2017	80	SPAEK-filled porous PAEK	DMFC	-	1.47	~55 (90°C)	~0.11 (90°C, 90% RH)	-
SPEEK-SrGO [41]	2017	-	Sulfonated reduced graphene oxide	PEMFC	1wt% SrGO	1.69	31.1 (80°C)	0.0086 (80°C 50%RH)	705 (70°C,80%RH, H_2_/Air)
BrPAEK-MeIm [35]	2018	-	Nitrogen-heterocycles	HTPEMFC	1.6 imidazole/unit	1.95	-	0.091 (170°C, 0% RH)	-
MeIm-PAEK/PVDF-HFP [36]	2018	-	MeIm-PAEK/PVDF-HFP blend	HTPEMFC	10% PVDF-HFP	-	103 (60°C)	0.219 (180°C, 0%RH)	-
SPEEK/Bu/SPEEK/Im [42]	2018	47	SPEEK/PU/SPEEK/bmim layer-by-layer	HTPEMFC	-	-	-	0.103 (160°C, 0%RH)	-
GO-g-SPEEK/Nafion-33 [30]	2018	80	GO, Nafion-33 blended	PEMFC	-	1.45	136.3 (90°C)	~0.23 (90°C)	213 (60°C, 50% RH, H_2_/Air)
XSPEEK/CNC [31]	2019	70	EG + CNC	PEMFC	67:33 (SPEEK:EG) 4wt% CNC	1.72	78.2 (95°C)	0.186 (95°C,95%RH)	-
SPEEK/PDA@PVDF [32]	2019	76.7	SPEEK embedded PDA-containing PVDF nanofibers	DMFC	85.7 wt% PDA@PVDF	-	~60 (80°C)	0.06 (80°C, 100%RH)	104 (5 M MeOH/O_2_, 70°C)
SSi-GO/SPEEK [33]	2019	-	SPEEK nanofibers/SSi-GO	DMFC	2.5wt%	1.65 (mmol/g)	~90 (70°C)	0.1566 (70°C,100% RH)	-
PAEK-b-KSPAEK/OSPN [34]	2019	-	PAEK-b-KSPAEK copolymer/OSPN	PEMFC	24wt% OSPN	-	84.01 (90°C)	~0.11 (90°C, 100%RH)	410 (80°C, 100%RH, H _2_/O_2_)

**Table 4:** Water uptake and electrochemical properties ofpolyimide(PI)-type PEMs.

PI-type									
Membrane	Year	%DS	Modifications	Fuel Cell Type	Filler Content	IEC(meq/g)	Water Uptake (%)	Proton Conductivity (S/cm)	Peak Power Density (mW/cm^2^)
SPI/FGO [43]	2015	-	Ionic liquid-functionalized graphene oxide	HTPEMFC	5wt% FGO	-	47.3	0.0772 (160°C, 40%RH)	-
CSiSPIBI [51]	2016	-	Silane-crosslinked sulfonated poly(imide benzimidazole)	HTPEMFC	60 mol fraction sulfonated diamine monomer	0.54	-	~0.1 (150°C,50%RH)	-
CSPI [44]	2017	-	Crosslinked SPI with pendant alkyl side chains containing trimethoxysilyl	DMFC	70 mol% DAPS groups	2.02 (mmol/g)	73.4 (80°C)	~0.13 (80°C,100%RH)	84.3 (2M MeOH/air, 60°C)
Aliphatic SPI [46]	2018	-	Aliphatic SPI with perylenediimide units	PEMFC	-	1.79 (mmol/g)	80 (80°C)	0.1864 (80°C, 100%RH)	931.88 (80°C, 100% RH, H_2_/O_2_)
NSPI [47]	2018	-	Novel SPI from NSDA/ODA	DMFC	50/50 (wt NSDA/wt ODA)	1.25	38.21 (35°C)		-
SPI-PE [48]	2018	-	SPI-PE charge transfer complex	PEMFC	0.33 molar ratio PE	2.16 (mmol/g)	45.9 (RT)	0.0201 (80°C, 90%RH)	~150 (80°C, 95%RH, H_2_/Air)
SPI Nanofiber framework [49]	2018	-	S-block graft (bg)-PI/S-r-PI nanofibers	PEMFC	80/20 (wt S-bg-PI/wt S-r-PI)	1.8	73.9 (RT)	>0.1 (80°C, 85%RH)	-
SPI-RHA [50]	2019	-	SPI-rice husk ash biofillers	Passive-DMFC	15 wt% RHA	0.2519 (mmol/g)	55.24	0.2058 (RT)	13 (2M MeOH, RT)

**Table 5:** Uptake and electrochemical properties of PES- and PSF-type PEMs.

PES- and PSF-type									
Membrane	Year	%DS	Modifications	Fuel Cell Type	Filler Content	IEC (meq/g)	Water Uptake (%)	Proton Conductivity (S/cm)	Peak Power Density (mW/cm^2^)
SPES-PBI [53]	2015	-	Ionic crosslinked with p-PBI	PEMFC	3wt% p-PBI	1.46	42.9 (80°C)	0.21 (80°C, 100%RH)	-
Imidazolium PSF [62]	2015	-	PSF with imidazolium pendants	HTPEMFC	-	-	-	0.04 (180°C, 0%RH)	269 (160°C, 0%RH, H_2_/O_2_)
SPES/CNW [54]	2016	-	Chitin nanowhiskers	PEMFC	7 wt% CNW	-	~19 (80°C)	~0.014 (80°C,100%RH)	-
SPES/NPHC [63]	2016	35	N-phythaloyl chitosan blend	DMFC	1wt% NPHC	1.29	41.5 (80°C)	0.0121 (80°C)	-
SPSF-SGO [64]	2017	71.55	Sulfonated graphene oxide	DMFC	3 wt% SGO	-	22.33	0.00427 (RT, 100%RH)	-
dsPFES-imPES[65]	2017	100	Sulfonated poly(fluorenyl ether sulfone)/imidazolium PES blend	PEMFC	2wt% imPES	1.17	89.7 (80°C)	0.35 (80°C,100%RH)	-
SPPSU/EG [55]	2018	2.24	SPPSU crosslinked with ethylene glycol (EG)	PEMFC	12 molecule EG/rpu	2.79	199 (RT)	0.23 (120°C,90%RH)	-
SPES-MOF [59]	2018	19	PES-SO_2_Cl/Cr-MIL-101-NH_2_ MOF	HTPEMFC	0.1 g MOF	3.18	35 (80°C)	0.041 (160°C, 0%RH)	238 (160°C, 0%RH, H_2_/O_2_)
PES-PVP [60]	2018	-	PES-PVP/graphitic carbon nitride (CN) nanosheets	HTPEMFC	0.5 wt% CN	-	-	0.12 (180°C, 0%RH)	634 (180°C, 0%RH, H_2_/O_2_)
SPES/S-MCM-41 [57]	2018	-	S-MCM-41 silica	PEMFC	2 wt% S-MCM-41	1.4	21.76	0.0694	-
SPSF/CW-Ser [58]	2019	40	Serine-modified cellulose nanowhiskers	DMFC	10wt% CW-Ser	-	~65 (80°C)	0.234 (80°C)	73.757 (60°C, 100%RH, 2M MeOH/O_2_)
SPSU/SPPSU [56]	2019	-	Multiblock copolymer SPSU/SPPSU	PEMFC	1:9 (PSU:TMSCS ratio)	1.58	31.2 (60°C)	0.025 (80°C, 95%RH)	400 (70°C,100%RH, H_2_/O_2_)
Am-SPAEKS/C-SPAEKS [61]	2019	-	Crosslinked SPAEKS with multiple sulfonic acid groups	PEMFC	2 molar ratio of AMPS to Am-SPAEKS-DBS	2.09	14.6 (80°C	0.135 (80°C, 100%RH)	121.09 (80°C,100%RH, H_2_/air)

**Table 6:** Uptake, acid doping level (ADL), and electrochemical properties of PBI-type PEMs.

PBI-type								
Membrane	Year	Modifications	Fuel Cell Type	Filler Content	PA Uptake (%)	ADL (PA/rpu)	Proton Conductivity (S/cm)	Peak Power Density (mW/cm^2^)
PBI-4BPO_x_[75]	2015	Boron phosphate	HTPEMFC	4 mole BPO_x_/mole PBI	-	-	0.045 (150°C,5%RH)	~500 (150°C,0%RH,H_2_/air)
Ph-PBI [76]	2016	Phenyl pendants	HTPEMFC	-	-	19.1 (160°C, 108 h)	0.138 (200°C,0%RH)	279 (160°C,0%RH,H_2_/air)
Me-PBI [76]		Methylphenyl pendants				17.6 (160°C, 108 h)	0.123 (200°C,0%RH)	320 (160°C,0%RH,H_2_/air)
PBIOH-ILS [77]	2017	Ionic liquid-functionalized silica	HTPEMFC	5% ILS	-	9.65 (110℃, 72 h)	0.106 (170°C,0%RH)	-
PBI-GO [78]	2017	Graphene oxide	HTPEMFC	2wt% GO	-	12 (336 h)	0.1704 (180°C, 0%RH)	380 (165°C, 0%RH, H_2_/Air)
P-b-O-PBI [73]	2018	p-PBI/OPBI multiblock copolymer	HTPEMFC	0.5:0.5 (p-PBI:OPBI)	-	7.9 (80°C)	0.1 (180°C, 0%RH)	360 (160°C,0%RH, H_2_/Air)
s-PBI [79]	2018	Azide naphthalene sulfonic acid-PBI	PEMFC	40wt% azide	-	-	0.006593 (RT, 0%RH)	-
PBI/lignin [80]	2018	Lignin	HTPEMFC	20wt% lignin	-	27 (RT, 24 h)	0.152 (160°C, 0%RH)	-
PBI-RGO/PPBI/PPBI-RGO [81]	2018	Radiation grafted sulfonated GO-PBI/Porous PB I three layer membrane	HTPEMFC	80% PPBI	500	20.4 (80°C, 48 h)	0.1138 (170°C, 0%RH)	-
g-PBI [70]	2018	Ph-PBI grafted with benzimidazolyl pendants	HTPEMFC	20% grafting degree	-	22.1 (120°C, 72 h)	0.212 (200°C, 0%RH)	443 (160°C, 0%RH, H_2_/O_2_)
ABPBI/S-Sep [82]	2019	Sulfonated sepiolite	HTPEMFC	2 wt% S-Sep	-	~3.5 (RT, 72 h)	0.051 (180°C, 0%RH)	230 (180°C, 0%RH, H_2_/O_2_)
cPBI-BF_4_[71]	2019	Crosslinked PBI with PBI-BuI PIL	HTPEMFC	40wt% PIL	362.5	19.7	0.117 (170°C, 0%RH)	-
CF6PBI-R2-6 [72]	2019	Crosslinked branched F6-PBI with BA-a	HTPEMFC	-	~69.5 (120°C)	-	~0.07 (180°C, 0%RH)	690 (160°C, 0% RH, H_2_/O_2_)
2,6-Py-PBI/PGO [73]	2019	Phosphonated graphene oxide	HTPEMFC	1.5 wt% PGO	-	5.8 (45°C, 168 h)	0.0764 (140°C, 0%RH)	359 (120°C, 0%RH, H_2_/Air)

**Table 7:** Water uptake, PA uptake, and the electrochemical properties of PPO-type PEMs.

PPO-type									
Membrane	Year	Modifications	Fuel Cell Type	Filler Content	IEC(meq/g)	Water Uptake (%)	PA Uptake (%)	Proton Conductivity (S/cm)	Peak Power Density (mW/cm^2^)
SPPO-HGM-SPPO [88]	2015	Hollow glass microspheres (HGMs)	DMFC	9wt% HGM	2.164	19.31	-	0.0318 (20°C, 100%RH)	81.5 (RT, 2M MeOH/O_2_)
PPO-MeIM[89]	2017	Methylimidazolium PPO	HTPEMFC	4:10 (MeIM:BPPO)	-	-	135 (30°C, 24 h)	0.0679 (160°C, 0% RH)	280 (160 °C, 0%RH, H_2_/O_2_)
SPEEK/BPPO [85]	2017	SPEEK/BPPO blend	DMFC	20wt% BPPO	1.21 (mmol/g)	11.76	-	0.064 (60°C, 100%RH)	23.9 (60 °C, 10M MeOH/O_2_)
SPPO+TEOS [84]	2017	TEOS-based silica nanoparticles	PEMFC	-	1.75	66	-	-	-
QPPO-MIm/ ATMP-APTES [90]	2019	Phosphonic acid-functionalized siloxane	HTPEMFC	15wt% ATMP-APTES	1.04 (mmol/g)	38.91 (80°C)	-	0.0848 (160°C, 5%RH)	638 (160 °C, 5%, H_2_/O_2_)
XTPPO [87]	2020	Crosslinked triazole PPO	HTPEMFC	40% bromination degree, 10% degree of crosslinking	-	-	211 (120°C, 15 h)	0.064 (180 °C, 0%RH)	-
